# Dietary Diversity in Pregnant Women and Its Association With Household Food Security in Rural Southern Angola

**DOI:** 10.1111/mcn.70051

**Published:** 2025-06-02

**Authors:** Rocio Martin‐Cañavate, Elena Trigo, Maria Romay‐Barja, Lourdes Maria Faria, Ana Silva Gerardo, Isabel Aguado, Eva Iráizoz, Tayna Marques, Israel Molina, Estefania Custodio

**Affiliations:** ^1^ Centro Nacional de Medicina Tropical, Instituto de Salud Carlos III Madrid Madrid Spain; ^2^ Escuela Internacional de Doctorado, Universidad Nacional de Educación a Distancia Madrid Madrid España; ^3^ Tropical Medicine Unit Vall d'Hebron‐Drassanes, Infectious Diseases Department, Vall d'Hebron University Hospital PROSICS Barcelona Barcelona Cataluña Spain; ^4^ Fundo Apoio Social ‐Local Development Institute, Avenida Pedro de Castro Vandunem Luanda Luanda Angola; ^5^ Faculdade de Medicina da Universidade Mandume Ya Ndemufayo, Bairro Comercial Lubango Huíla Angola; ^6^ Action Against Hunger Spain Madrid Madrid Spain; ^7^ Centro de Investigación Biomédica en Red de Enfermedades Infecciosas, (CIBERINFEC), Instituto de Salud Carlos III Madrid Madrid Spain

**Keywords:** Angola, dietary diversity, food insecurity, Food Insecurity Experience Scale (FIES), Household Dietary Diversity Score (HDDS), pregnant women

## Abstract

Dietary diversity in pregnant women is key for maternal health and newborn growth and development. Food insecurity is one of the determinants of poor quality diets and can be measured by several indicators, but evidence of its association with dietary diversity in pregnant women is limited. We assessed the effect of food insecurity measured by the Food Insecurity Experience Scale (FIES) and by the household dietary diversity score (HDDS) on pregnant women's dietary diversity score in four rural areas of South Angola, using the baseline survey data of the MuCCUA trial (“Mother and Child Chronic Undernutrition in Angola” study). We constructed separate estimation models for each food insecurity indicator and adjusted for confounders on the overall sample and by “commune”. Among 1379 pregnant women, only 6.7% met the minimum dietary diversity for women, 78.3% of the households experienced moderate/severe food insecurity as measured by FIES and 73.8% showed food insecurity reflected by a low HDDS. Significant negative effects were found between food insecurity measured by low HDDS and pregnant women's dietary diversity after adjusting for confounders, in all the models. Experiencing moderate/severe food insecurity as measured by FIES (FIESmodsev) also had a negative effect on pregnant women's dietary diversity overall, but the effect was only maintained in two communes. Results were similar for severe food insecurity (FIESsev) but effect was lost after adjusting for confounders in three communes. Although low HDDS, FIESmodsev and FIESsev showed similar prevalences of food insecurity, their impact on pregnant women's dietary diversity was different in terms of strength and significance. Addressing food insecurity should be considered when designing multifaceted interventions for improving dietary diversity in pregnant women.

**Trial registration:** Clinical Trials NCT05571280. Registered 7 October 2022.

## Summary

- The study reveals a critical food crisis in rural communities of South Angola, as evidenced by alarming levels of food insecurity and poor nutrition indicators.
- Only 7% of pregnant women met the minimum dietary diversity required for a healthy pregnancy, reflecting widespread nutritional vulnerability.
- Household food insecurity affected nearly three in four households, 73.8% showed low HDDS and 78.3% were classified as moderately or severely food insecure according to FIES.
- The effect of food insecurity on the women's diets differed by the indicator used to measure it‐ low HDDS consistently predicted poorer diets, while FIES measures showed more variable associations.

## Graphical abstract

Only 7% of pregnant women in rural South Angola reached the minimum dietary diversity for women. Food insecurity measured by Food Insecurity Experience Scale and by the Household Dietary Diversity Score was above 70%, but the association with women dietary diversity varied depending on the indicator used.

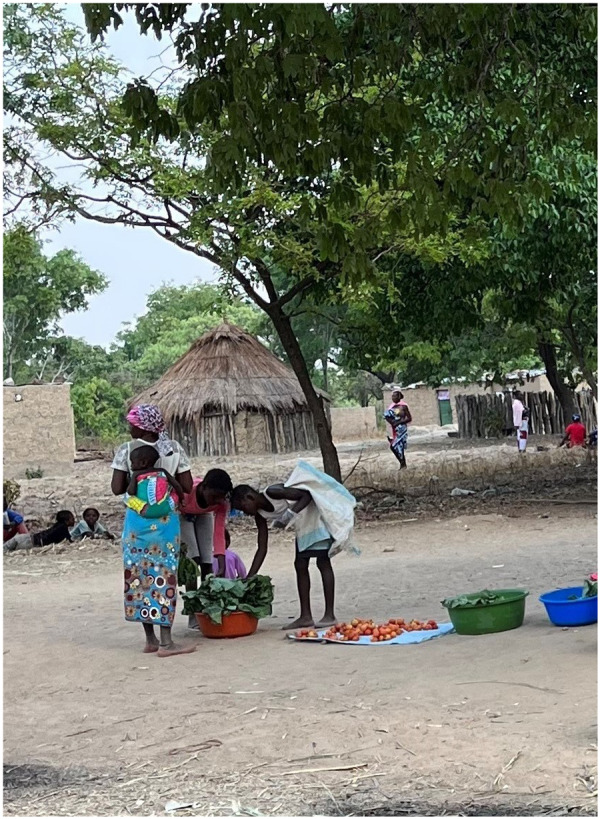

## 1 Introduction

Adequate nutrition during pregnancy is essential to promote both maternal health and newborn growth and development because of increased nutritional requirements during this period. Insufficient micronutrient intakes can affect women and infants health, development and survival especially during the first 1000 days of life (Torheim and Arimond 2013). One of the important dimensions of diet quality is diet diversity that measures the number of different food groups consumed at household or individual level. The minimum dietary diversity for women of reproductive age (MDD‐W) is an indicator developed as a proxy for micronutrient adequacy in women of this age group (FAO 2021), and validated for its use in pregnant women (Verger et al. 2024).

Food insecurity is one of the determinants of low dietary diversity among women (Johnson et al. 2018; Na et al. 2016). Food insecurity exists when people lack physical and economic access to safe and nutritious foods that meet their dietary needs and food preferences for an active and healthy life (FAO et al. 2024). People experiencing severe food insecurity are unlikely to be able to acquire enough food to continuously fulfil their dietary energy and micronutrient requirements (FAO, IFAD, UNICEF, WFP and WHO 2020). Various indicators are available to measure food insecurity as they reflect different food security dimensions: availability, access, utilization and stability (Manikas et al. 2023). The Household Dietary Diversity Score (HDDS) is a proxy indicator meant to reflect the economic ability of a household to access a variety of foods (Swindale and Bilinksy 2006). The food groups used in calculating the HDD score reflect the general accessibility of these foods and the households purchasing power rather than the nutritional quality of the diet (Kennedy et al. 2012). Other food security indicators collect data with different methodologies and varied recall periods, such as the Household Hunger Scale and the share of income dedicated to food. Other indicators are experience‐based and measure the construct of food insecurity, such as the Household Food Insecurity Access Scale (HFIAS) or the Latin American and Caribbean Food Security Scale (ELCSA). The HFIAS measures both access and experience of severity of household food insecurity during the last 30 days (Coates et al. 2007), and it is one of the most widely used experience‐based indicator for food insecurity assessment (Manikas et al. 2023). Lastly, the Food Insecurity Experience Scale (FIES) indicator, developed in 2017 by the FAO‐Voices of the Hungry project, was introduced in the Sustainable Development Goals (SDGs) to measure SDG#2. It assesses access to food by asking people directly about their experience at different levels of severity that can be compared across contexts (Cafiero et al. 2018). The prevalence of moderate/severe food insecurity based on FIES is one of the indicators to track progress toward reaching SDGs goal 2.1.2, which aims to end hunger and ensure yearn‐round access by all people to safe, nutritious and sufficient food by 2030 (FAO, IFAD, UNICEF, WFP and WHO 2023). One of the distinctive contributions of the FIES and similar experienced‐based food insecurity measures is that they not only consider compromised diet quality and reduced food quantity, but also capture psychosocial elements associated with anxiety or uncertainty regarding the ability to procure enough food, a feature that other non‐experience‐based indicators do not (Food and Agricultural Organization 2016).

Globally, the prevalence of moderate/severe food insecurity (SDG indicator 2.1.2) is estimated to be 28.9 percent in 2023, which translates into a total of 2.3 billion people. However, it has been reported that worldwide food insecurity disproportionately affects women, reflecting persistent gender inequalities and rural populations (FAO et al. 2024). Likewise there are studies documenting that women consume less diverse diets than their households (Gupta et al. 2020) (Harris‐Fry et al. 2018), and that cultural practices and beliefs especially during pregnancy can influence dietary diversity through specific dietary restrictions and taboos (Das and Mishra 2021), often leading to nutritional deficiencies (Chakona and Shackleton 2019). To our knowledge, no published data exists on women's dietary diversity in Angola nor on pregnant women. Although the effect of food insecurity on women's dietary diversity has been previously documented in the literature, the variety of food security indicators used hinders the comparison of results (Johnson et al. 2018). Most studies found a negative association between nonpregnant women's dietary diversity and different indicators of food insecurity (increasing food insecurity diminishes women's dietary diversity), though some found no association. A study in Niger found that women's dietary diversity score (WDDS) declined with lower HDDS and in households experiencing hunger as measured by the Household Hunger Scale (Cisse‐Egbuonye et al. 2017). Moreover, several studies in low‐resource settings have formerly found significant negative associations between household food insecurity measured by HFIAS and dietary diversity among women of reproductive age in Bangladesh (Harris‐Fry et al. 2015), Somalia (Mohammed et al. 2023), Burkina Faso (Custodio et al. 2020), and Nepal (Singh et al. 2020). However, studies in Tanzania (Huang et al. 2018) using the HFIAS to measure food insecurity and in the United States using the Standard Food Security Survey Module (Gamba et al. 2016) did not find any significant association between household food insecurity and women's dietary diversity. Yet, little evidence exists on the association between food insecurity as measured by FIES and women's dietary diversity. Only one study including Syrian refugee mothers in Lebanon indicated that higher food insecurity levels measured by FIES were significantly correlated with lower dietary diversity but did not adjust for confounding factors (Abou‐Rizk et al. 2022). In contrast, another study in Ethiopia assessing factors associated with pregnant women's dietary diversity found no association between MDD‐W and food insecurity as measured by FIES (Getahun et al. 2023). In addition to the contradictory evidence, few studies have examined this association in pregnant women, a particularly vulnerable population to food insecurity and its negative impacts because of their increased nutritional requirements to support foetal growth and maternal health, especially in resource‐limited settings (Augusto et al. 2020) (McKay et al. 2022). Only three studies in Malawi using HFIAS (Kang et al. 2019), in Bangladesh using the Food Access Survey Tool (Na et al. 2016) and in Ghana using the Food Consumption Score (Saaka et al. 2021) for food security assessment showed that food‐insecure pregnant women had a lower dietary diversity score compared to food‐secure pregnant women.

Limited studies used the FIES indicator, recommended for tracking SDG 2.1.2, to explore the effect of food insecurity on women's dietary diversity (Abou‐Rizk et al. 2022), with only one study focusing on pregnant women and finding no association (Getahun et al. 2023). Moreover, very few studies compared different food insecurity indicators within the same sample (Manikas et al. 2023). Given the unique nature of pregnancy, experience‐based food security scales have been shown to perform differently for pregnant women (Hromi‐Fiedler et al. 2009). Consequently, our findings would contribute to a better understanding of how different food insecurity scales perform in this population, as well as to address the lack of published data on women's dietary diversity in Angola. Therefore, in the present study we aimed to assess how food insecurity relates to pregnant women's dietary diversity using two different indicators, one based in access to food and one experience‐based, in a severely food insecure area in Southern Angola. The objectives of the study are:

– To assess the dietary diversity and food insecurity situation of pregnant women in two Southern provinces of Angola.
– To estimate the effect of food insecurity measured by two different indicators (FIES and HDDS) on the dietary diversity of these pregnant women.

## 2 Methods

### 2.1 Study Setting and Population

According to UNICEF, 7.3 million people in Angola face food and nutrition insecurity (Angola Humanitarian Situation Report 2022). Recurrent drought, reduced agricultural production, crop failures and generalized rise in food prices are particularly compromising maternal and infant nutrition. The Southwestern provinces of Cunene, Huila and Namibe are the most affected with high malnutrition rates and food security crises, particularly rural areas, according to the latest Integrated Food Security Phase Classification (*Angola: Acute Food Insecurity Situation and Acute Malnutrition Situation April 2021 ‐ March 2022* | *IPC Global Platform* 2022). In this context, the FRESAN (Fortalecimento da Resiliência e da Segurança Alimentar e Nutricional em Angola) program was launched in 2018 through a bilateral partnership between the European Union (EU) and the Government of Angola. This EU‐funded program aims at strengthening the resilience and food and nutrition security in Southern Angola. One of its components is the Crescer Project, an operational research programme on the prevention of chronic malnutrition in Huila and Cunene provinces (*Crescer – Pesquisa Operacional Contra a Desnutrição Crónica Infantil Em Angola* n.d.). The Crescer project implements the MuCCUA trial (“Mother and Child Chronic Undernutrition in Angola” study), a three‐arm community trial of the cost‐effectiveness of three multi‐sectoral interventions to prevent chronic malnutrition during the first 1000 days in Southern Angola.

The trial is set in the two provinces of Huila and Cunene. A total of two communes per province were included: the commune of Libongue and the commune of Jamba in the province of Huila; and the commune of Mupa and the commune of Otchinjau in Cunene province meeting the inclusion criteria (Custodio et al. 2024). According to the latest census, in 2014, the total population of Huila province was 3,185,244 and Cunene province was 1,271,638. They are predominantly rural and highly dependent on staple foods (maize, millet and sorghum), but differences in livelihoods exist between them. Huila has higher agricultural production and lower food insecurity risk compared to Cunene, which relies mostly on livestock activity and where the semiarid conditions and frequent droughts occasionally result in livestock loss (Martin‐Cañavate et al. 2023).

The MuCCUA study population are pregnant women and their newborn children living in the four communes of Jamba, Libongue, Mupa and Otchinjau, which are target recipients of either arm: standard of care (SOC), SOC + nutrition supplementation, or SOC + unconditional cash transfer. A total of 36 clusters were randomized to include 1440 pregnant women who are being followed until their newborns are 2 years of age. The analysis presented in this study used baseline survey data of MuCCUA trial participants, based on a cross‐sectional pre‐intervention questionnaire. The MuCCUA trial methodology has been previously published (Custodio et al. 2024) (Martin‐Cañavate et al. 2023).

### 2.2 Data Collection

The baseline survey was conducted from October 2022 to March 2023. Out of 1440 pregnant women planned for recruitment, a total of 1423 pregnant women aged 16–49 years were recruited and surveyed. Pregnancy was confirmed by a positive pregnancy test performed during the recruitment process coinciding at the time of the survey. The questionnaires collected general household information, along with specific information of the household head, the primary caregiver and the participant pregnant women. We collected information on the household's socio‐demographic and asset data, food security, knowledge, attitudes and practices related to children's caring practices as well as hygiene and sanitation, women's empowerment, dietary diversity at household, women and children's level, perinatal care and pregnant women's morbidity and mental health.

Pilot studies were conducted both in Huila and Cunene provinces for fieldwork training and tools testing. Original questionnaires were in Portuguese and translation to local languages was conducted during the training with enumerators. The questionnaires were administered using a digital case report form (e‐CRF) within the Ennov Clinical app, developed for trial support by the Clinical Research Organization Bioclever. Data were collected on smart devices running Apple‐based platforms.

### 2.3 Variables Construction

#### 2.3.1 Women's Dietary Diversity

Women dietary diversity score (WDDS) was calculated as a proxy of diet quality using data from the 24‐h recall of the food groups consumed by participant pregnant women based on the Diet Quality Questionnaire (DQQ) for Angola (Global Diet Quality Project 2022). Pregnant women were asked to carefully describe everything they ate or drank during the previous 24 h inside and outside the house. Answers were registered and food items were then aggregated in the 10 predefined food groups: 1‐Grains, white roots and tubers; 2‐Pulses; 3‐Nuts and seeds; 4‐Dairy; 5‐Meat, poultry and fish; 6‐Eggs; 7‐Dark green leafy vegetables; 8‐Other Vitamin A‐rich fruits and vegetables; 9‐Other vegetables; 10‐Other fruits (FAO 2021). Minimum dietary diversity for women (MDD‐W) is a dichotomous indicator of whether women 15–49 years of age have consumed at least 5 out of 10 defined food groups in the last 24 h. MDD‐W variable was constructed by computing 1 if the women had consumed five or more food groups, and 0 if food groups consumed were below five.

#### 2.3.2 Food Insecurity

Food security was measured by two different indicators: HDDS and FIES. Overall food insecurity (including moderate and severe) was defined as having a low household dietary diversity score or experiencing moderate/severe food insecurity based on the FIES.

#### 2.3.3 Household Dietary Diversity Score (HDDS)

To measure food insecurity by household dietary diversity, data were collected using a 24‐h recall method asking the person who cooked food in the household about food groups consumed in the household (Swindale and Bilinksy 2006) (Kennedy et al. 2012). This study includes foods eaten by any member of the household, and exclude foods purchased and eaten outside the home. The value of the Household Dietary Diversity Score variable for a household ranges from 0 to 12 for food consumed by members of the household (1 = Yes, 0 = No). Then, scores were categorized in two levels, considering food insecurity as a low HDDS with 0–4 food groups and food security as a high HDDS with 5–12 food groups consumed by members of the household (IPC Global Partners 2021).

#### 2.3.4 Food Insecurity Experience Scale (FIES)

To measure food insecurity by moderate and severe FIES, the FIES, is collected with an 8‐item questionnaire, which asks participants to self‐report about their experience of food insecurity as a measure of peoples access to food (Food and Agricultural Organization 2016). A 12‐month reference period was used as recommended for SDG monitoring. Respondents answered yes/no to (1) being worried about not having enough food, (2) being unable to eat healthy and nutritious food, (3) eating only a few kinds of food, (4) to skip a meal or (5) ate less, (6) ran out of food, (7) were hungry but did not eat, (8) went without eating for a whole day. Responses to the eight dichotomous questions were coded 1 = Yes and 0 = No. Rasch modelling (Bond 2015) was applied to analyze FIES data as recommended by FAO, following the principles of Item Response Theory (FAO 2016) (Ballard et al. 2013). Study population prevalence of food insecurity at moderate and severe levels was estimated after statistical validation on the quality of the FIES data collected following FAO guidelines (FAO 2016). The Rasch reliability value was 0.71, meaning that the FIES fulfilled the Rasch model assumptions of conditional independence and equal discrimination, while also meeting the fit statistic standards for all eight items. Probabilities of moderate/severe food insecurity (FIESmodsev) and of severe food insecurity (FIESsev) were categorized in dichotomous variables for regression analyses using the rule of a probability < 0.5 as food secure, and a probability > =0.5 as food insecure for both levels of insecurity.

#### 2.3.5 Socioeconomic Score (SES)

Socioeconomic status of the household was assessed using principal component analysis (Jolliffe and Cadima 2016) with information collected on the following variables: ownership of household assets (motorbike, wagon, hoe, machete, motor bomb, plough, tractor, car, mobile phone, radio, TV, gas or electric cooker and generator); any land ownership; any livestock ownership and number of large, medium and small animals. To reflect the cluster nature of the data, a data‐driven approach was used to classify households using cluster analysis (Cortinovis et al. 1993). Score distributions were heavily skewed to the right because the level of homogeneity between households was high (Schellenberg et al. 2003). The reason for this was that one of the inclusion criteria to enter the trial was to have a multidimensional poverty level of 4 or 5 according to the Angola National Statistical Institute classification (Instituto Nacional de Estatística de Angola et al. 2020) at the municipality level (Custodio et al. 2024). For this reason and to better classify households into SES groups, we used cluster analysis on the household socioeconomic score derived for each commune to classify them into ‘low’, ‘medium’ and ‘high’ socioeconomic groups (Vyas and Kumaranayake 2006).

#### 2.3.6 Other Variables

Household‐ and individual‐level characteristics were assessed and explored as potential confounding factors, including pregnant women age, education, gestational age, age at first pregnancy and gravidity, head of household age, sex and education, having soap and having suffered any shock in the last 6 months.

### 2.4 Statistical Analysis

The data were cleaned by deleting the records of individual women without dietary data collected. For the analysis, a total of 1379 records were included out of 1423.

Descriptive statistics are presented as frequency and percentage for categorical variables and as mean and standard deviation for continuous variables. We conducted bivariate analyses to assess differences in household characteristics and food group's consumption across communes.

We first performed bivariate logistic regression analyses with MDD‐W as a dichotomous outcome variable and low HDDS, FIESmodsev and FIESsev as independent variables separately on the overall sample of pregnant women. We then explored the association of covariates with each of the indicators to identify those associated to both outcome and independent variables. Only variables that showed a significant association at the *p* < 0.05 level were selected to be introduced in the multivariable models.

We then developed three separate estimation models to assess the effect of (1) low HDDS, (2) moderate/severe food insecurity measured by FIESmodsev and (3) severe food insecurity measured by FIESsev on the dietary diversity of pregnant women, independently. We computed three multivariable logistic regression models using as outcome variable the MDD‐W adjusted for confounding factors and accounting for village‐level clustering. Results were reported as unadjusted odds ratio (unadj. OR), adjusted odds ratio (adj.OR) with 95% confidence intervals (95%CIs) and *p*‐value.

Using WDDS as a continuous outcome variable, we carried out bivariate and multivariate lineal regression models separately with (1) low HDDS, with (2) FIESmodsev and then with (3) FIESsev, in the overall sample and for each of the communes accounting for village‐level clustering. Results were reported as unadjusted beta coefficient (unadj.β), adjusted beta coefficient (adj.β) with 95%CIs), and *p*‐value.

Potential confounding variables selected for inclusion in the adjusted analysis were based on existing literature on possible related factors to WDDS including socioeconomic status, women age and education, head of household age, sex and education, gravidity, having soap and suffering shocks in the last 6 months. Correlation among confounding variables was checked before their inclusion in the final regression models. The final multivariate models included all variables that were significant for each of the overall and commune's models if the crude and adjusted effect differed by > 10%, and that did not show correlation values between them above 0.65. We also tested for interaction terms with each of the covariates. P‐values less than 0.05 were considered statistically significant. STATA software version 17.0 was used for all statistical analyses.

### 2.5 Ethical Considerations

The research protocol of the MuCCUA trial was approved on August 18, 2022 by the National Ethics Committee of República de Angola, Ministério da Saúde, reference number 27 C.E/MINSA. INIS/2022, and all methods were performed according to guidelines of the Helsinki Declaration. The trial was registered in *clinicaltrials.gov* in October 7, 2022, NCT05571280.

## 3 Results

### 3.1 General Characteristics of Study Population

A total of 1379 pregnant women and their households were assessed (Table 1). The mean age of participants was 26.4 years and 47% were younger than 25 years. Half of the women (49.7%) were in the second pregnancy trimester, and 65.5% of the women were already mothers of three or more children. More than half of the participants had primary education or higher (52.6%), being the prevalence being higher than 52% in all communes except for Otchinjau where only 15% had primary education or higher. Most of the households (68.4%) were headed by males and the mean household size was 6.6 ± 6.2 (SD).

**Table 1:** Sample characteristics stratified by commune.

	Overall	Jamba	Libongue	Mupa	Otchinjau
	*N* = 1379	*N* = 357	*N* = 349	*N* = 330	*N* = 343
**Outcome variables**					
Women's dietary diversity score (WDDS)	2.58 (1.30)	3.18 (1.17)	3.16 (1.11)	2.28 (1.16)	1.66 (1.09)
Minimum Dietary Diversity Women (MDD‐W)					
< 5	93.3	89.1	89.4	97.3	98.0
> =5	6.7	10.9	10.6	2.7	2.0
**Independent variables**					
Household Dietary Diversity Score (HDDS)					
Low HDDS (0–4 food groups)	73.8	57.7	62.7	84.2	91.8
High HDDS (5–12 food groups)	26.2	42.3	37.3	15.8	8.2
Moderate/severe food insecurity (FIESmodsev)					
Moderate/severe food insecurity > 0.5	78.3	74.5	81.8	67.0	89.3
Food secure < =0.5	21.7	25.5	18.2	33.0	10.7
Severe food insecurity (FIESsev)					
Severe food insecurity > 0.5	64.6	53.5	65.9	53.5	85.5
Food secure < =0.5	35.4	46.5	34.1	46.5	14.5
**Household level characteristics**					
Socioeconomic score					
Low	36.3	50.7	34.1	12.1	46.9
Medium	47.2	37	55.3	53.7	43.5
High	16.5	12.3	10.6	34.2	9.6
Household youth ratio score (mean)	0.23	0.19	0.21	0.26	0.25
Age of head of household					
16–20	6.5	9.5	10.0	2.0	4.1
21–35	52.7	54.1	61.3	40.9	53.1
35–55	40.8	36.4	28.7	57.1	42.8
Sex of head of household					
Male	68.4	68.6	71.5	68.6	64.7
Female	31.6	31.4	28.5	31.4	35.3
Head of household education					
No education	45.4	27.1	34.0	36.8	84.5
Primary or higher	54.6	72.9	66.0	63.2	15.5
Head of household same participant woman					
No	76.0	77.2	76.8	80.3	71.0
Yes	24.0	22.8	23.2	20.7	29.0
Person who decides on food					
The children's mother	76.4	75.7	74.0	75.1	80.7
The father or someone else	23.6	24.3	26.0	24.9	19.3
**Individual‐level characteristics**					
Women age					
15–24	47.4	52.1	56.2	35.8	44.9
25–34	36.6	37	31.5	41.5	36.7
35–49	16.0	10.9	12.3	22.7	18.4
Gestational age					
1st trimester	20.5	13.6	23.8	22.9	22.2
2nd trimester	49.7	48.3	51.2	47.7	51.8
3rd trimester	29.8	38.1	25.0	29.4	26.0
Any prenatal visit during this pregnancy					
No	28.9	23.2	24.8	31.1	36.8
Yes	71.2	76.8	75.2	68.9	63.2
Gravidity					
0–2 pregnancies	34.4	44	31.5	31.5	30.4
3 or more pregnancies	65.5	56	68.5	68.5	69.6
Age at first pregnancy					
15–17 years	42.6	55.6	58.5	23.5	30.5
18–20 years	40.6	34.8	34.7	51.1	42.5
> 21 years	16.8	9.7	6.8	25.4	27
Education					
No education	47.4	33.7	39.3	33.2	84.9
Primary or higher	52.6	66.3	60.7	66.8	15.2
Woman treats drinking water					
No	48.8	44.4	39	49.5	62.5
Yes	51.2	55.6	61	50.5	37.5
Has soap at home					
No	36.1	23.5	26.9	67.9	62.8
Yes	63.9	76.6	73.1	32.1	37.2

### 3.2 Pregnant Women Dietary Diversity and Food Group Consumption

The mean of the dietary diversity score for pregnant women was 2.58 ± 1.30 (SD). Of the 1379 women, only 6.7% met the MDD‐W. The percentage was significantly higher in Jamba and Libongue (10.9% and 10.6% respectively), and lower in Mupa (2.7%) and Otchinjau (2.0%) (*p* < 0.001). In Table 2, we can observe the overall differences in food groups' consumption by MDD‐W status. Among overall pregnant women who met the MDD‐W and consumed at least five different food groups, the most common combination of food groups was grains, dark green leafy vegetables, other vegetables, meat, poultry and fish and pulses. There were statistically significant differences between pregnant women reaching the MDD‐W and those not reaching it in the consumption of meat, poultry and fish (73.9% vs. 26.0%), eggs (20.7% vs. 2.1%) and other vegetables (85.9% vs. 22.0%).

**Table 2:** Percentage of food groups consumed categorized by minimum dietary diversity score results in overall sample of pregnant women (*n* = 1379).

	MDD > = 5 (n = 92)	MDD < 5 (n = 1287)
Food groups	%	%
Grains, white roots and tubers, and plantains	100.0	97.4
Dark green leafy vegetables	90.2	56.3
Other vegetables	85.9	22.0
Meat, poultry and fish	73.9	26.0
Pulses (beans, peas and lentils)	67.4	16.0
Other fruits	46.7	7.5
Other vitamin A‐rich fruits and vegetables	33.7	1.9
Milk and milk products	22.8	6.8
Eggs	20.7	2.1
Nuts and seeds	13.0	0.9

**Figure 1:**
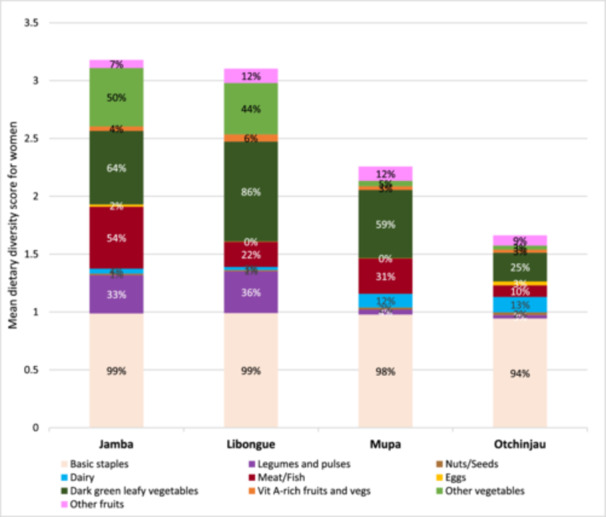
Mean dietary diversity and food group's consumption in pregnant women across two communes in Huila (Jamba and Libongue) and two communes in Cunene (Mupa and Otchinjau), Angola.

**Figure 2:**
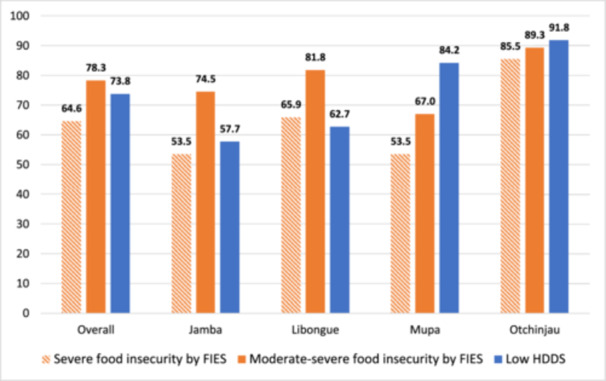
Prevalence of food insecurity measured by FIESmodsev, FIESsev and HDDS in pregnant women across two communes in Huila (Jamba and Libongue) and two communes in Cunene (Mupa and Otchinjau), Angola.

The differences in food group's consumption between communes were marked as shown in Figure 1. Legumes and pulses consumption was significantly higher in Jamba (33%) and Libongue (36%) than in Mupa (4%) and Otchinjau (3%). In contrast, dairy products were significantly more frequently consumed in Mupa (12%) and Otchinjau (13%) as compared to the 4% and 3% in Jamba and Libongue. For all four communes, eggs consumption was below 3% and vitamin A rich fruits and vegetables below 6%. Figure 2.

### 3.3 Food Insecurity

Overall prevalence of food insecurity measured by low household dietary diversity score was 73.8%, being significantly higher in Mupa (84.2%) and Otchinjau (91.8%) than in the other two communes (*p* < 0.001). The mean HDDS of the total sample was 3.36 ± 1.82 (SD) ranging from 4.19 ± 1.65 (SD) in Jamba to 2.04 ± 1.59 (SD) in Otchinjau.

Among pregnant women's households, the overall proportion experiencing moderate/severe food insecurity as measured by FIES was 78.3%, while the proportion experiencing severe food insecurity was 64.6%. Proportions of severe food insecurity were significantly lower in Jamba and Mupa (53.5%), compared to Libongue (65.9%) and Otchinjau where it was the highest of all communes (85.5%) (*p* < 0.001).

#### 3.3.1 Estimation Models Results

Tables 3 and 4 report results of unadjusted and adjusted effects of low HDDS, FIESmodsev and FIESsev separately on pregnant women's dietary diversity and confounding factors of the effects.

**Table 3:** Regression results. Effect of food insecurity as measured by either FIES or low HDDS on MDD‐W and WDDS overall and by commune.

	Low household dietary diversity score (Low HDDS)		Moderate/severe food insecurity (FIESmodsev)		Severe food insecurity (FIESsev)	
	OR (CI: 95%)	*p*	OR (CI: 95%)	*p*	OR (CI: 95%)	*p*
MDD‐W overall						
Unadjusted	0.069 (0.041, 0.118)		0.327 (0.208, 0.513)		0.320 (0.203, 0.503)	
Adjusted	0.117 (0.060, 0.228)	< 0.00	0.463 (0.261, 0.822)	0.009	0.477 (0.273, 0.832)	0.009

	**β (CI: 95%)**	***p***	**β (CI: 95%)**	***p***	**β (CI: 95%)**	***p***
WDDS overall						
Unadjusted	−1.619 (−1.750, −1.488)		−0.564 (−0.731, −0.397)		−0.683 (−0.825, −0.542)	
Adjusted	−1.292 (−1.448, −1.135)	0.0001	−0.351 (−0.546, −0.156)	0.001	−0.413 (−0.538, −0.287)	0.0001
WDDS‐Jamba						
Unadjusted	−1.101 (−1.319, −0.883)		−0.937 (−1.215, −0.659)		−0.699 (−0.947, −0.452)	
Adjusted	−1.029 (−1.245, −0.813)	0.000	−0.848 (−1.239, −0.456)	0.001	−0.557 (−0.871, −0.242)	0.004
WDDS‐Libongue						
Unadjusted	−1.371 (−1.566, −1.176)		−0.284 (−0.591, 0.219)	0.069	−0.421 (−0.668, −0.174)	
Adjusted	−1.371 (−1.566, −1.176)	0.000	—	—	−0.306 (−0.613, 0.002)	0.051
WDDS‐Mupa						
Unadjusted	−1.221 (−1.540, −0.903)		−0.161 (−0.416, 0.094)	0.216	−0.338 (−0.577, −0.100)	
Adjusted	−1.209 (−1.580, −0.839)	0.000	—	—	−0.267 (−0.543, 0.008)	0.056
WDDS‐Otchinjau						
Unadjusted	−1.807 (−2.184, −1.429)		−0.620 (−0.985, −0.255)		−0.452 (−0.773, −0.131)	
Adjusted	−1.640 (−2.491, −0.789)	0.002	−0.256 (−0.467, −0.044)	0.023	−0.097 (−0.345, 0.151)	0.403

MDD‐W, Minimum dietary diversity for women (dichotomous variable). For MDD‐W, results are reported as odds ratio with 95% CI. WDDS, Women dietary diversity score (continuous variable). For WDDS, results are reported as beta coefficient and 95% CI.

**Table 4:** Confounding factors of the association between pregnant women dietary diversity and food insecurity as measured by low HDDS, FIESmodsev and FIESsev.

	Confounding factors						
	Commune	Socioeconomic status	Education	Having soap	Gravidity	Age	Shocks last 6 monhts
MDDW overall							
Low HDDS	—	NS	+	+	—	NS	NS
FIESmodsev	NS	+	+	+	NS	NS	NS
FIESsev	NS	+	+	+	NS	NS	NS
WDDS overall							
Low HDDS	—	NS	NS	NS	NS	NS	NS
FIESmodsev	—	+	+	+	NS	NS	—
FIESsev	—	NS	+	+	NS	NS	NS
WDDS‐Jamba							
Low HDDS	N/A	NS	—	NS	NS	NS	NS
FIESmodsev	N/A	NS	NS	+	NS	NS	NS
FIESsev	N/A	+	NS	+	NS	NS	NS
WDDS‐Libongue							
Low HDDS	N/A	NS	NS	NS	NS	NS	NS
FIESmodsev	N/A	N/A	N/A	N/A	N/A	N/A	N/A
FIESsev	N/A	+	NS	+	NS	NS	NS
WDDS‐Mupa							
Low HDDS	N/A	NS	NS	NS	NS	—	NS
FIESmodsev	N/A	N/A	N/A	N/A	N/A	N/A	N/A
FIESsev	N/A	NS	NS	NS	—	NS	NS
WDDS‐Otchinjau							
Low HDDS	N/A	+	NS	NS	NS	NS	NS
FIESmodsev	N/A	+	NS	+	NS	NS	—
FIESsev	N/A	+	+	+	NS	NS	—

Abbreviations: ESmodsev, moderate/severe food insecurity as measured by Food Insecurity Experience Scale; FIESsev, severe food insecurity as measured by Food Insecurity Experience Scale; HDDS, Household Dietary Diversity Score; MDD‐W, Minimum dietary diversity for women; N/A, not applicable; NS, not statistically significant; WDDS, Women dietary diversity score; +, Positive significant association; −, Negative significant association.

Overall, there was a statistically significant association between low HDDS and MDD‐W. Compared to pregnant women with a higher HDDS, pregnant women with a low HDDS were less likely to meet the MDD‐W (adj. OR: 0.12; 95%CI: 0.06–0.23) after adjusting for commune, education, having soap and gravidity.

Pregnant women living in households categorized as moderate/severe food insecurity measured by FIESmodsev were less likely to reach the MDD‐W, compared to pregnant women living in households categorized as food secure (unadj. OR: 0.32; 95%CI: 0.21–0.51). After adjusting for confounding factors (women educational level, SES and having soap), see Table 4, the magnitude of the effect was attenuated but still significant (adj. OR: 0.46; 95%CI: 0.26–0.82). These results were very similar in pregnant women experiencing severe food insecurity (FIESsev).

We explored the impact of low HDDS, FIESmodsev and FIESsev on women's dietary diversity scores as a continuous variable. Analyses were performed in the overall sample and in each of the communes. In line with MDD‐W, the impact of low HDDS on WDDS for the overall sample was stronger (adj.β: −1.292; 95%CI: −1.448, −1.135) than the effect of FIESmodsev (adj. β: −0.351; 95%CI: −0.546, −0.156) and FIESsev on WDDS (adj.β: −0.413; 95%CI: −0.538, −0.287). All three were negatively associated with WDDS after adjusting for confounders (*p* < 0.01). It is worth noting that several confounding factors affected the association in both FIESmodsev and FIESsev models, but not in the low HDDS model.

Results were disaggregated by communes as commune was identified as a confounder and modifier in the association between the food insecurity indicators and the women's dietary diversity score in the overall sample (see Table 4).

Among pregnant women from Jamba, Libongue, Mupa and Otchinjau, having a low HDDS was significantly associated with lower WDDS (−1.029, −1.371, −1.209 and −1.640 lower scores respectively), compared to pregnant women with a high HDDS. No covariates were found to be confounding factors of this association in Jamba, Libongue and Mupa. Only SES was a confounder in Otchinjau slightly attenuating the negative effect of low HDDS on WDDS (unadj.β: −1.807 vs. adj.β: −1.640).

In relation to food insecurity measured by FIES, the bivariable analyses showed that there was not a significant association between FIESmodsev and WDDS in the communes of Libongue or Mupa. In contrast, in Jamba and Otchinjau there was a significant negative effect of FIESmodsev on WDDS both in bivariate and multivariate models. In Jamba the negative effect remained almost the same after adjusting for having soap (unadj.β: −0.937 vs. adj.β: −0.848), whereas in Otchinjau the negative effect was weakened after adjusting for SES, having soap and having suffered a shock in the last 6 months (unadj.β: −0.620 vs. adj.β: −0.256).

The significant negative effect of severe food insecurity measured by FIESsev on WDDS observed in the bivariate analysis only remained significant for Jamba (unadj.β: −0.699 vs. adj.β: −0.557) after adjusting for SES and having soap (*p* = 0.004). In Libongue, the negative effect of FIESsev on WDDS found in the bivariate analysis was lost after adjusting for SES and having soap (adj.β −0.306; 95%CI: −0.613, 0.002; *p* = 0.05). The same happened for Mupa after adjusting for gravidity (*p* = 0.056). In Otchinjau the negative effect of severe food insecurity (FIESsev) on WDDS disappeared and lost significance (*p* = 0.403) after adjusting for SES, pregnant women education, having soap and having suffered shocks in the last 6 months (unadj.β: −0.452 vs. adj.β: −0.097). See Tables 3 and 4.

## 4 Discussion

Overall, the minimum dietary diversity for women of the MuCCUA trial was extremely low, with only 7% of the pregnant women reaching the MDD‐W. In addition, the total proportion of households experiencing moderate/severe food insecurity was alarmingly high: 73.8% as measured by low HDDS and 78.3% according to FIES, which also indicated that 64.6% of households were experiencing severe food insecurity.

These results highlight a severe food insecurity crisis among the poorer communities of these rural areas in South Angola. According to Integrated Food Security Phase Classification (IPC), a standardized tool used to analyze and classify the severity of food insecurity and malnutrition in a given region for decision‐making, the overall sample of this study would be classified in a Crisis Phase 3. This reflects that urgent action is required to protect livelihoods and reduce food consumption gaps (IPC Global Partners 2021). Moreover, Otchinjau commune would be classified as Emergency Phase 4 reflecting that inmediate intervention is imperative to save lives and livelihoods because household have large food consumption gaps. The increased food insecurity in Otchinjau, classified as Phase 4, may be attributed to the commune's unique conditions, including greater household dispersion and remoteness compared to the other three communes, limited accessibility to public services, and the absence of phone and internet access. This aligns with the latest Famine Early Warning Systems Network (FEWS NET) report, which classified the provinces of Huila and Cunene in Crisis (IPC Phase 3) and in need of humanitarian food assistance in 2023 (FEWS NET 2023). The dry weather conditions in Southern Angola during the 2022/2023 season significantly reduced agricultural production, the primary food source for rural households. In addition, persistent food inflation further constrained food access, exacerbating the acute food insecurity in the region (FSIN and Global Network Against Food Crises 2024). In Sub‐Saharan Africa, other countries like Liberia (71.5%) and South Sudan (87.3%) have also reported very high severe food insecurity estimates measured by FIES, but these have been largely attributed to their political instability (Wambogo et al. 2018).

The low MDD‐W (7%) also reflects this food crisis, highlighting the monotonous diets of pregnant women in these areas, where the mean of the women's dietary diversity score was 2.6, with diets primarily based on grains and dark green leafy vegetables. These results are concerning as pregnancy is a particular period when nutritional requirements increase. Other physiological factors related to the uniqueness of pregnancy might influence dietary diversity such as cravings and morning sickness (Placek 2018). Pregnant women's dietary diversity could also be influenced by cultural beliefs and traditions (Das and Mishra 2021), such as imposing specific dietary restrictions and taboos, which often result in nutritional deficiencies (Chakona and Shackleton 2019). The proportion of pregnant women reaching the MDD‐W was particularly low in the communes of Cunene province (Mupa and Otchinjau). The majority of Cunene's population consists of agro‐pastoralists groups who rely primarily on cattle and limited subsistence farming in a semi‐arid climate. While meat consumption was low, dairy consumption was higher compared to Huila. This might be due to the cultural significance of livestock, which is rarely slaughtered for family consumption or exchanged for food, as their value is predominantly tied to social prestige (Ribeiro and Satiaca 2021). As a result, livestock products like dairy are consumed, but not the meat. In contrast, Huila communities are predominantly agrarian, which is reflected in our findings where women from Jamba and Libongue consumed more dark leafy green vegetables and other vegetables than those from Cunene.

In relation to the food security indicators, our findings show that the prevalence of severe food insecurity measured by Food Insecurity Experience FIES was consistent with the prevalence of food insecurity measured by a low Household Dietary Diversity Score (HDDS). However, the prevalence of moderate/severe food insecurity varied between the FIES and HDDS, depending on the commune analyzed. For example, in Mupa, the prevalence of moderate/severe food insecurity was lower according to FIES (67.0%) compared to HDDS (84.2%). Conversely, in Libongue, the prevalence of moderate/severe food insecurity was higher with FIES (81.8%) than with HDDS (62.7%). These discrepancies highlight the importance of exploring how the recently incorporated FIES compares with other food insecurity indicators such as Household Dietary Diversity Score, HFIAS and Household Hunger Scale, in different contexts.

In addition, we found mixed results in the association between household's food insecurity and the dietary diversity of pregnant women depending on the food insecurity indicator used. Food insecurity measured by a low HDDS showed a consistently significant negative association with the dietary diversity of pregnant women, greater food insecurity was linked to lower dietary diversity. Conversely, the association between moderate/severe food insecurity measured by the FIES and dietary diversity was significant in the overall sample and in some of the communes, but not universally.

The findings align with existing literature. For example, a study in Niger found that a unit increase in HDDS corresponded to a 0.396 increase in WDDS having a positive significant effect (Cisse‐Egbuonye et al. 2017). Other research in lower‐middle‐income countries showed similar results using the HFIAS, one of the most commonly used experience‐based food insecurity indicator (Manikas et al. 2023). In Malawi, Burkina Faso, Bangladesh and Pakistan, women's dietary diversity was consistently negatively associated with food insecurity as measured by HFIAS (Kang et al. 2019) (Na et al. 2016; Custodio et al. 2020) (Hashmi et al. 2021).

In contrast, the literature on the association between the FIES and women's dietary diversity is limited (Manikas et al. 2023). One study of Syrian refugee mothers found a moderately strong negative correlation between WDDS and severe food insecurity as measured by FIES (Abou‐Rizk et al. 2022), though it did not adjust for confounding factors. Another study in Ethiopia did not find an association between FIES and dietary diversity among pregnant women (Getahun et al. 2023).

Moreover, the association between low HDDS and WDDS was only confounded by household socioeconomic status in one commune (Otchinjau), whereas multiple factors confounded the association between FIES and WDDS across all communes. Higher socioeconomic status and women with at least primary education were protective against the impact of food insecurity on dietary diversity. Our findings are consistent with other studies indicating that higher socioeconomic status and education improve access to diverse food and diet quality for women in resource‐poor settings (Bandyopadhyay et al. 2021; Harris‐Fry et al. 2015).

The stronger and more consistent association between low HDDS and WDDS compared to FIES may be due to differences in the dimensions and time periods assessed by each indicator (Manikas et al. 2023). That is HDDS and WDDS are both based on food groups consumption and are highly correlated as the availability of diverse food within the household significantly influences individual foods consumption (Gupta et al. 2020). The HDDS reflects economic access to food based on household‐level food group consumption over the previous day, while women's dietary diversity is measured individually, and also based on food groups consumption the day before. In contrast, FIES assesses subjective food insecurity experiences during the past year, which may limit its ability to show significant associations with women's dietary diversity.

Given that FIES is a recommended indicator for tracking progress towards SDG Goal 2.1.2 Ending Hunger and Malnutrition, further research is needed to clarify its relationship with other food insecurity indicators and with dietary diversity.

### 4.1 Strengths and Limitations

This study was narrowed to the baseline sample of the MuCCUA trial, that is implemented in extremely vulnerable communities (Custodio et al. 2024). Therefore, estimates of MDD‐W and food insecurity of this study are not representative at administrative level. The cross‐sectional nature of the study does not allow establishing causality between the studied variables, neither to consider the impact of seasonality in the results. Nevertheless, our study provides valuable information, as to our knowledge, no data on women dietary diversity has been published for Angola. In addition, the pregnant women of this sample are part of the MuCCUA longitudinal randomized community trial and are being followed with their newborns until 2025 when the children will turn 2 years of age. Thus, we will be able compare the results by season. A final strength of the study was the use of two alternative food security indicators, HDDS and FIES (SDG#2 indicator) to assess their effect on women's dietary diversity score separately, because limited evidence is available.

## 5 Conclusion

The elevated food insecurity estimates measured by both HDDS and FIES place this population in Southern Angola at Crisis Phase (IPC3) according to international standards, reflected also in the extremely low dietary diversity of the pregnant women studied. To improve MDD‐W in the four communes, women could include more pulses, vegetables, meat, poultry and fish in their diets whenever possible, as these are the food groups most consumed by pregnant women that reached the MDD‐W in our sample.

Experiencing moderate/severe food insecurity as measured by low HDDS and FIES had a negative impact on pregnant women's dietary diversity score and MDD‐W. The two indicators used to measure food insecurity drew similar estimates of food insecurity, but their association with the pregnant women's dietary diversity was different. Food insecurity measured by low HDDS was consistently associated with low pregnant women's dietary diversity in all contexts while food insecurity measured by FIES was only associated in some of them and with less strength. This may be associated to the fact that FIES and MDD‐W have very different recall periods and data collection methods.

As the use of FIES indicator is recommended for SDG#2 surveillance, and the measurement of women's dietary diversity is highly recommended in contexts of food insecurity, we encourage further studies to look at how they relate to each other. Policy makers and programme implementers might consider multifaceted interventions to enhance women food security and dietary diversity especially during pregnancy and lactation to improve maternal and newborn health and nutrition outcomes.

## Author Contributions

R.M.‐C. and E.C. contributed to the conception and design of the study and drafted the manuscript. E.T., M.R.‐B., I.A., L.M.F., A.S.G., E.I., T.M., I.M. and E.C. contributed to the conception and design of the MuCCUA trial protocol in which this study is embedded. E.T., I.A., L.M.F. and A.S.G. led and supervised all intervention implementation activities and contributed to field data acquisition. All authors read, edited and approved the final manuscript.

## Ethics Statement

The research protocol of the MuCCUA trial was approved on August 18, 2022 by the National Ethics Committee of República de Angola, Ministério da Saúde, reference number 27 C.E/MINSA.INIS/2022, and all methods were performed according to guidelines of the Helsinki Declaration.

## Consent

Pregnant women's informed consent was obtained for their own participation and for their potential newborn children's in the MuCCUA trial. Further details are available in the published protocol (Custodio et al. 2024).

## Conflicts of Interest

The authors declare no conflicts of interest.

## Data Availability

The data that support the findings of this study are available on request from the corresponding author. The data are not publicly available due to privacy or ethical restrictions. The data that support the findings of this study are available from the project coordinator, Israel Molina Romero, on reasonable request at israel.molina@vallhebron.cat.
